# Enhanced hybrid hydrogel based on wheat husk lignin-rich nanocellulose for effective dye removal

**DOI:** 10.3389/fbioe.2023.1160698

**Published:** 2023-03-15

**Authors:** Rong Huang, Yong Xu, Boris N. Kuznetsov, Meitao Sun, Xin Zhou, Jing Luo, Kankan Jiang

**Affiliations:** ^1^ School of Basic Medical Sciences and Forensic Medicine, Hangzhou Medical College, Hangzhou, China; ^2^ Jiangsu Co-Innovation Center of Efficient Processing and Utilization of Forest Resources, College of Chemical Engineering, Nanjing Forestry University, Nanjing, China; ^3^ Institute of Chemistry and Chemical Technology SB RAS, FRC KSC SB RAS, Siberian Federal University, Krasnoyarsk, Russia

**Keywords:** dye, adsorption, hybrid hydrogel, lignin-rich nanocellulose, wheat husk

## Abstract

Polyvinyl alcohol (PVA) hydrogels were enhanced mechanically through the addition of lignin-rich nanocellulose (LCN), soluble ash (SA) and montmorillonite (MMT) for dye removal. The hybrid hydrogels reinforced with 33.3 wt% of LCN had a 163.0% increase in storage modulus as compared to the PVA/0LCN-33.3SM hydrogel. LCN can be added to the PVA hydrogel to alter its rheological properties. Additionally, hybrid hydrogels were highly efficient in removing methylene blue from wastewater, which was attributed to the synergistic effects of the PVA matrix supporting embedded LCN, MMT, and SA. The adsorption time (0–90 min) showed that the hydrogels containing MMT and SA had high removal efficiency, and the adsorption of methylene blue (MB) by PVA/20LCN-13.3SM was greater than 95.7% at 30°C. It was found that MB efficiency decreased with a high MMT and SA content. Our study provided a new method for the fabrication of polymers-based eco-friendly, low-cost and robust physical hydrogels for the MB removal.

## 1 Introduction

The uncontrolled releasing of organic dyes into the environment has caused a variety of hazards to humans (Mohammed et al., 2014; Tang et al., 2020). There are several technologies developed for the treatment of hazardous organic dyes from wastewater in order to address this issue (Ge et al., 2022a). The most commonly used technologies are membrane filtration, chemical precipitation, ion exchange, and electrolysis. However, there are several disadvantages associated with these technologies, including their expensiveness, ineffectiveness, and the emission of secondary wastes. Hence, a better approach (i.e., adsorption) could be a cheaper and more effective way to treat organic dyes (El-Dib et al., 2020; Obey et al., 2022).

Biosorbents produced from lignocellulosic biomass like wood and agricultural wastes have recently drawn considerable attention (Freitas et al., 2019). In general, the lignocellulosic biomass particularly those derived from agricultural wastes, can be used to yield a wide range of versatile products at low costs and with substantial sustainability (Han et al., 2015; Chen et al., 2019). Wheat husk (WH), an agricultural by-product abundant throughout the world that contains high amounts of carbohydrates (25.1% cellulose, 21.4% hemicellulose, and 20.5% lignin), could serve as a promising resource for producing biosorbents for adsorption. Direct combustion of WH results in significant resource waste due to its inefficiency (Ge et al., 2021; Ge et al., 2022b; Liu et al., 2022). In order to maximize the use of lignocellulosic biomass, the hemicellulose of WH is used to prepare xylose-based products such as oligosaccharides (XOS). XOS is composed of a low degree of polymerization of xylan (DP = 2–7) (Otieno and Ahring, 2012). XOS production involves a variety of methods (chemical, enzymatic and auto-hydrolysis). Auto-hydrolysis has been reported as an effective and practical method to produce XOS because of reduced catalyst costs (Carvalho et al., 2013).

When hemicellulose is removed from the WH residue in the XOS production process, the cellulose content of the residue is high (50%–55%). These residues can be used for the preparation of microcrystalline cellulose and nanocellulose. Nanocellulose is produced from cellulose. Most of the literature reports purity cellulose obtained from agricultural residues by chemical pretreatment. In fact, the aim of these treatments is to remove lignin, however, consuming large amounts of chemicals (e.g., sodium hydroxide and sodium chlorite) (Xiao et al., 2022). Therefore, it is conjectured that the production of lignin-containing nanocellulose (LCN) can mitigate environmental issues and difficulties in alkali recovery. Additionally, a low-cost and environmentally friendly pretreatment can also be used to produce LCN directly from WH residues (Bian et al., 2019; Liu et al., 2021). The lignin found in plant biomass may be useful as a binder for other biopolymers and may serve as a component in the production of hydrogels. In addition, since lignin is rich in the active chemical functional groups thus its presence would enhance the adsorption ability of the material (Liu et al., 2020; Gao et al., 2021).

Hydrogel is one of the important materials that widely studied in adsorption research. Hydrogel represents a hydrophilic, highly cross-linked and three-dimensional structure material that has excellent chemical properties. As a result of the presence of hydroxyl groups in each repeating molecular unit of polyvinyl alcohol (PVA), the polymer can form physically cross-linked hydrogels. Despite its adsorption and mechanical properties, hydrogels themselves have poor adsorption and mechanical properties that limit their application in adsorption (Lu et al., 2017; Luo et al., 2021). Although the combination of LCN and PVA may be a potential solution to improve the mechanical properties of PVA hydrogels (Bian et al., 2018). Unfortunately, the adsorption performance of this hydrogel is not promising. Hence, a filler is commonly required to improve the adsorption performance of the hydrogel.

To improve the mechanical properties and adsorption capacity of a hydrogel, we have constructed a composite hydrogel using montmorillonite (MMT) and the soluble ash (SA) as fillers followed by combination with PVA to explore the possibility of such approach. The SA was obtained by washing WH with water to potentially improve its adsorption capacity for organic dyes in the sewage field (Zhou and Wang, 2010; Wu et al., 2019). MMT is phyllosilicate clay; it has a three-dimensional porous structure and a large specific surface area, thus has high adsorption performance (Mao et al., 2022). Moreover, the mechanical properties of the resulted hydrogels can be enhanced which was attributed to the high mechanical strength of MMT (Luo et al., 2021). To the best of our awareness, there are limited literatures on the effects of various SA and MMT contents on the adsorption performance and physical strength of PVA/LCN/SA/MMT hydrogels (PLSM). Therefore, our results would devote to the sum of knowledge in this field while also revealing a new hydrogel based on wheat husk as a cheaper and eco-friendly material for dye removal (Zhang et al., 2022).

## 2 Materials and methods

### 2.1 Experimental materials

Montmorillonite (MMT) and 99% hydrolyzed polyvinyl alcohol (PVA) with a molecular weight of 125,000 g/mol were procured from Sigma-Aldrich. Wheat husk (WH) was supplied by Shandong Province, China. To remove the insoluble and soluble ash, the raw material was rigorously cleaned for 30 times using distilled water. The soluble ash was collected by rotary evaporation. The washed WH was treated for 60 min at 180°C to obtain XOS and the WH residue (WHR). The LCN (1 wt%) was produced from the WHR through a UH-20 homogenizer at 200 bar (Union-Biotech, China) (Luo et al., 2020).

### 2.2 Preparation of PLSM hydrogels

About 1.0 g of SA plus 1.5 g of MMT were poured into deionized water (50 mL) under stirring and yielding the 5.0 wt% mixture (i.e., SM). The composite aerogels were prepared by mixing LCN, PVA, and SM. The PVA loading was always fixed at 66.7%, while the LCN loading was 0, 3.0, 10.0, 20.0 and 33.3 wt% with the remaining amount of SM. The control aerogel was prepared by PVA. The total content of the mixture was kept at 4 wt% for all hydrogels (Table 1).

**TABLE 1:** Preparation parameters for PLSM hybrid hydrogels.

Sample	PVA:LCN:SM wt%	Mass ratio of PVA:LCN:SM
PVA	100	1
PVA/0LCN-33.3SM	66.7/0/33.3	1:0:0.50
PVA/3LCN-30.3SM	66.7/3/30.3	1:0.04:0.45
PVA/10LCN-23.3SM	66.7/10/23.3	1:0.15:0.35
PVA/20LCN-13.3SM	66.7/20/13.3	1:0.30:0.20
PVA/33.3LCN-0SM	66.7/33.3/0	1:0.50:0

### 2.3 Characterizations of PLSM hydrogels

The structural characteristics of LCN were measured with a Dimensiona Edge atomic force microscopy (AFM) manufactured by Bruker in Germany. The AFM images were resulted using a tip with a curvature radius of 8 nm and 300 kHz tapping mode. A Quanta 200 scanning electron microscope (SEM) manufactured by FEI in United States was adopted to examine the surface characteristics of PLSM hydrogel. Before the SEM analysis, the moisture of the samples was minimized followed by sputter-coated with gold to ensure it conducted electricity during analysis. A PerkinElmer Fourier Transform infrared (FTIR) spectrometer operating at wavenumber of 600–4,000 cm^−1^ was applied on the hydrogel samples to study the chemical functional groups. An X-ray diffractometer was utilized to obtain the diffraction patterns of the hydrogels over the 2θ value up to 35°. Additionally, the CrI, which is commonly known as crystallinity index, was then quantified using Eq. 1, consistent with the standard approach (Segal et al., 1959):

$$CrI=\frac{I_{200}-I_{am}}{I_{200}}\times 100$$ (1)

Where I_200_ was the maximum peak intensity of approximately 22.4° at a 2θ angle and I_am_ was the intensity of 18.4° at a 2θ angle.

The hybrid hydrogel was discarded from the 15 × 11 mm sample holder and moisture content was removed at 105°C for 24 h. The water content (W) of the hybrid hydrogel was measured with a decrease in mass according to Eq. 2:

$$W=\frac{m_{0}-m_{1}}{m_{0}}\times 100$$ (2)

The initial mass (g) of the hydrogel was m_0,_ while the mass of the treated hydrogel was m_1_.

An RS6000 rheometer (HAAKE, Germany) was used to measure the rheological behavior of the hydrogel with a cone plate P20 TiL and Platte P20 TiL. Then, the samples were processed into cylindrical shape with dimension of 20 mm (diameter) and 1 mm (thickness) (Liu et al., 2016).

### 2.4 Adsorption study of methylene blue (MB) using prepared hydrogels

Adsorption experiments were performed on MB (0.25 mg/mL) at different temperatures in a water bath at 80 rpm. A 0.5 g of the adsorbent dosage was gently mixed to the prepared MB solution (30 mL). Upon completion of the adsorption period, the treated MB solution was sampled into a 0.5 µm syringe. The concentrations after adsorption were immediately quantified at a wavelength of 663 nm using an UV-1800 UV-spectrometer (Japan). Finally, the amount of MB adsorbed was determined according to Eq. 3. All measurements were triplicated with common statistics calculated.

$${\;Q}_{t}=\frac{V\left(C_{0}-C_{e}\right)}{M}$$ (3)

C_0_ (before) and C_e_ (after) denote the concentration of the MB solution (mg/L), M (g) represents the adsorbent amount used in adsorption, and V (mL) represents the solution volume.

## 3 Results and discussion

### 3.1 Characterization of the prepared PVA/LCN/MMT/SA

**FIGURE 1:**
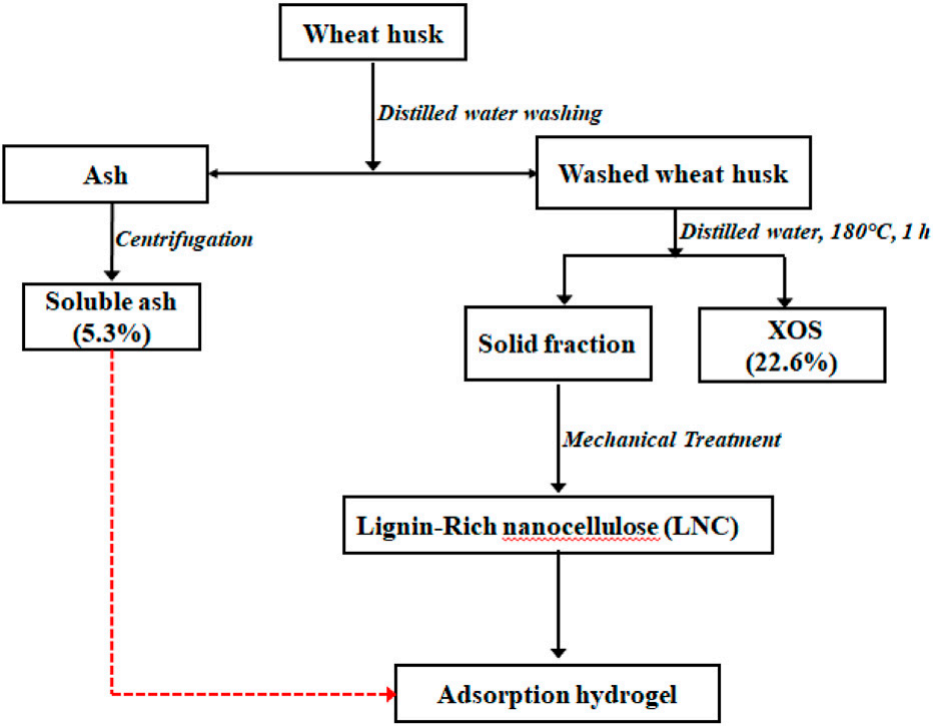
Flow diagram of the preparation of soluble ash (SA) and lignin-containing nanocellulose (LCN) of wheat husk.

**FIGURE 2:**
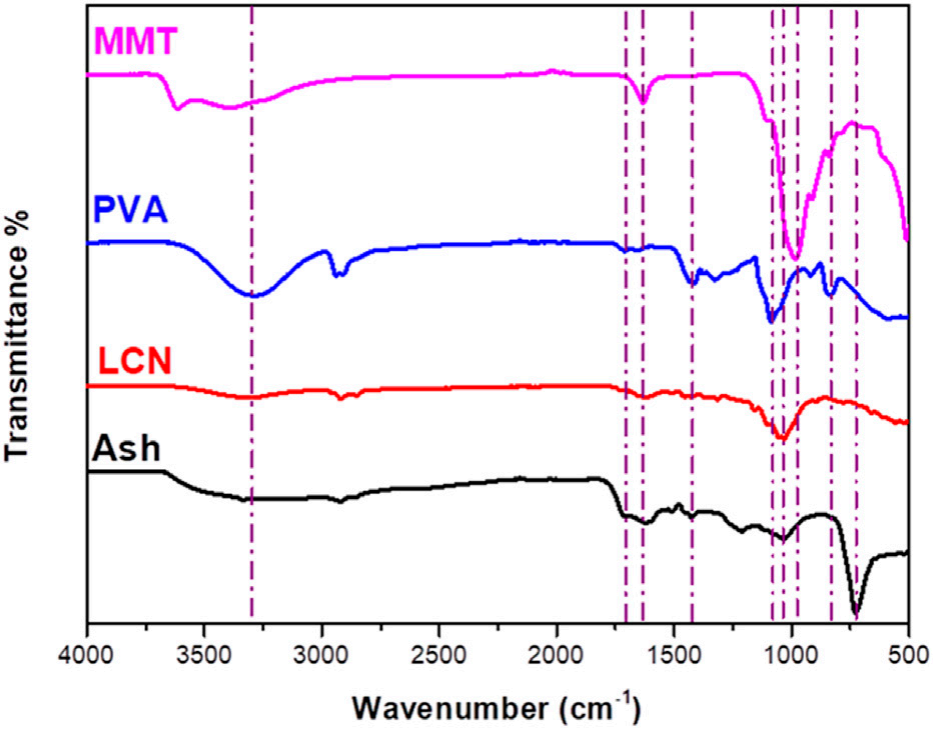
FTIR spectra of PVA/LCN/MMT/SA.

SA represents a mixture of inorganic and organic contents with good sorption properties of organic pollutants (Wu et al., 2020). The yield of SA recovered using distilled water treatment was 5.3% (calculated based on the washed ash). 5.0 g of the washed WH was pretreated with 50 mL of distilled water at temperatures (180°C) for 1 h. The XOS yield quantified based on the initial xylan content in the washed WH was 22.6%. The yield of lignin-containing nanocellulose (LCN) obtained from high-pressure homogeneous dispersion of WH residue (WHR) was 99.2% (Figure 1). The FTIR spectra of PVA/LCN/MMT/SA are displayed in Figure 2. The major peaks at 3,614, 1,612, 993 and 910 cm^−1^ of MMT were attributed to the stretching vibrations of OH, Si-O-Si and Al-OH, respectively (Tao et al., 2020). The bands of PVA occurring at 832, 921, 1,085, 1,424 and 3,400 cm^−1^ corresponded to the C−O stretching, −CH_2_ bending, −CH rocking, –CH and -OH stretching, respectively. The FTIR spectra showed some characteristic peaks that were correlated to the C-OH stretching bonds in the cellulose at 1,033 (secondary alcohol) and 1,052 cm^−1^ (primary alcohol) (Lam et al., 2017). The peak of methoxy group can be assigned at 1,470–1,430 cm^−1^ while the peak of C=C extension of the aromatic hydrocarbon can be found at 1,600–1,460 cm^−1^. Both peaks in these regions represented the characteristic peaks commonly found in lignin. The carboxyl stretching group was observed around 1732 cm^−1^ in the SA. Then, the Si-O-Si stretching vibration of silicate minerals peak was found at 1,034 cm^−1^. For the absorption region below 1,000 cm^−1^ is mainly the organic-mineral mixed zone. In this region, the characteristic peaks of different substances interfere with each other and overlap, thus there is no practical reference value (Matamala et al., 2019).

**FIGURE 3:**
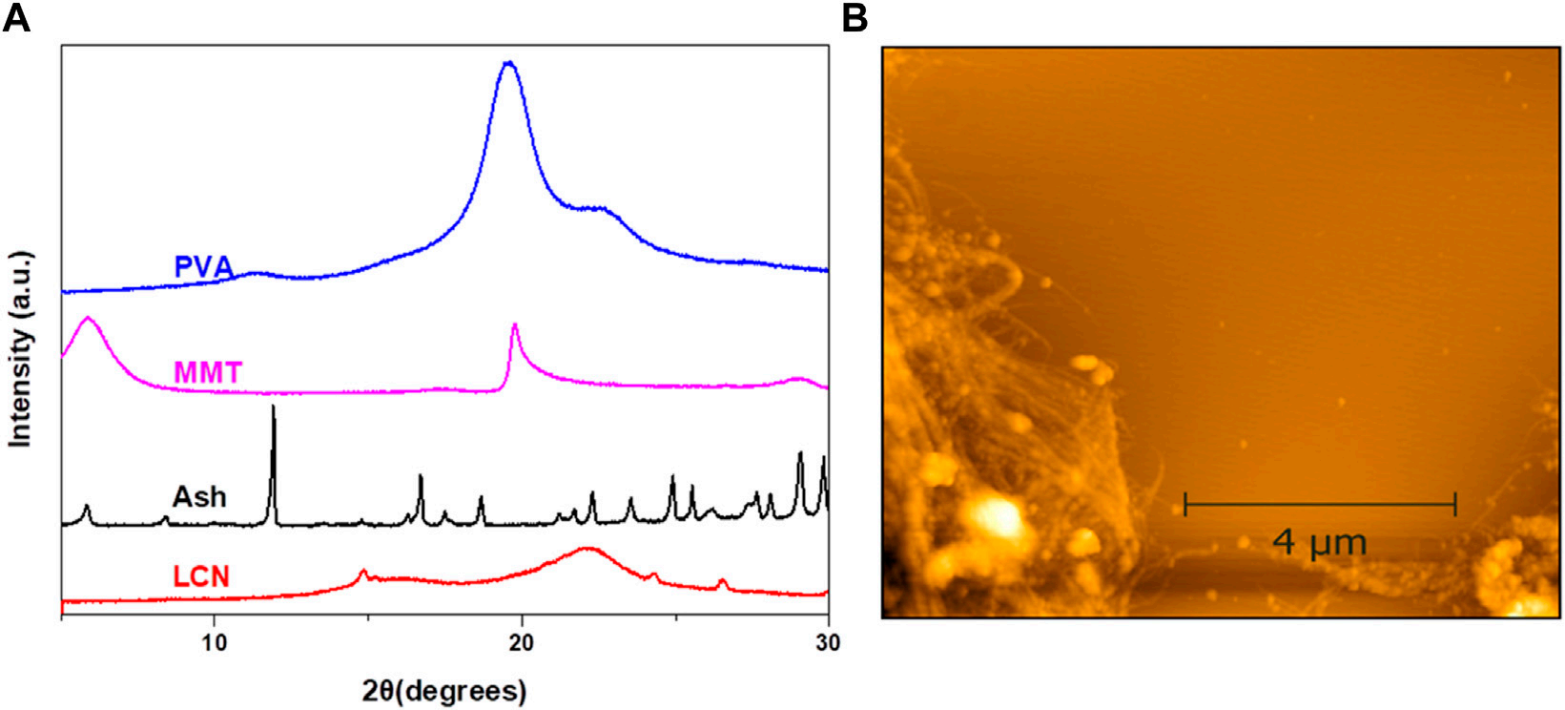
**(A)** XRD patterns of PVA/LCN/MMT/SA; **(B)** AFM image (Scale bar = 1 μm) of LCN.

The crystallinity of PVA/LCN/MMT/SA was examined *via* the XRD pattern of each sample displayed in Figure 3A. Based on the results, pure PVA had an orthogonal lattice structure, typical of semi-crystalline PVA, with a diffraction peak at 19.6° (Han et al., 2014). An XRD spectrum of MMT showed the characteristic peak at 6.37°. The XRD spectra of LCN showed two peaks at 16.1° and 22.5°, corresponding to the trigonal structure of cellulose I (Figure 3) with 49.9% crystallinity (Jia et al., 2017). The result indicated that amorphous cellulose was more prone to fracture than crystalline cellulose. The XRD spectra of SA inferred the presence of metal elements (K^+^, Na^+^, Ca^2+^, Mg^2+^) that have a good adsorption effect on MB (Wu et al., 2019). The AFM image showed nanofibers with small widths and narrow size distribution. The result displayed that mechanical treatment was an effective method to prepare LCN with a higher aspect ratio (Figure 3B).

**FIGURE 4:**
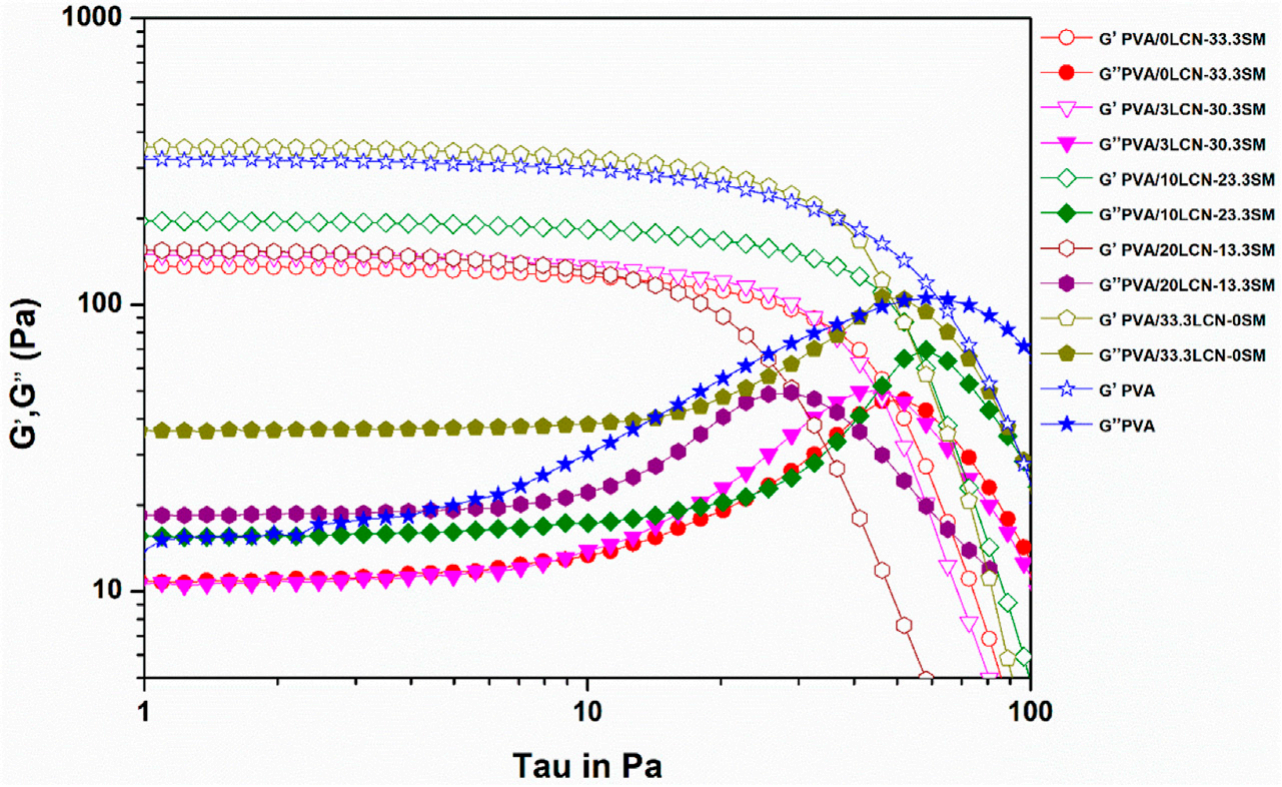
Stress scanning of loss modulus (G″) and shear storage modulus (G′) of the hydrogel reinforced by LCN at different loadings.

Figure 4 displayed the viscoelasticity tests of PLSM hydrogel at 25°C. Elasticity and viscosity are attributed to storage modulus (G′) and loss modulus (G″), respectively. The loss modulus (G″) of the PLSM hydrogel was lower than the storage modulus (G′), indicating the prepared hydrogels had regular hydrogel characteristics. The hydrogel flow and stress resistance can be determined by the location and length of the linear viscoelastic regions. The G′ of PLSM hydrogels was 136.0, 146.5, 156.5, 194.3 and 357.7 Pa when LCN was added at 3%, 10%, 20% and 33.3% (Figure 4), respectively. The G′ increased greatly with the increase of LCN dosage. This could be due to the LCN fibrils being long and thin (according to the average height of AFM), which promotes fibril entanglement. Therefore, adding LCN to PVA hydrogels could improve their rheological and mechanical properties (Chang et al., 2008). Comparing pure PVA hydrogels with those containing SM, Table 2 indicated SM decreased rheological and mechanical properties. This may be due to the low molecular weight of SM that reduced the interaction forces between the polymers. PLSM hydrogels with high levels of LCN possess excellent mechanical properties and are therefore suitable for making biomaterials without cross-linking. In addition, the property of having metallic elements on the hydrogels can greatly expand their applications.

**TABLE 2:** Physical, rheological and adsorptive properties of the hydrogels at various LCN loadings.

Samples	Moisture content (wt%)	Maximum G′ with LVR, G′ max (Pa)	Maximum G″ with LVR, G″ max (Pa)	Pseudo-first-order kinetic at 40°C (*R* ^2^)	Pseudo-second-order kinetic at 40°C (*R* ^2^)
PVA	95.4	325.5	14.2	—	—
PVA/0LCN-33.3SM	94.7	136.0	10.4	0.85	0.81
PVA/3LCN-30.3SM	94.8	146.6	10.6	0.98	0.98
PVA/10LCN-23.3SM	94.8	156.5	15.3	0.78	0.98
PVA/20LCN-13.3SM	94.6	194.3	18.4	0.64	0.98
PVA/33.3LCN-0SM	94.8	357.7	36.1	0.88	0.98

### 3.2 Characterization of the PLSM hydrogels

**FIGURE 5:**
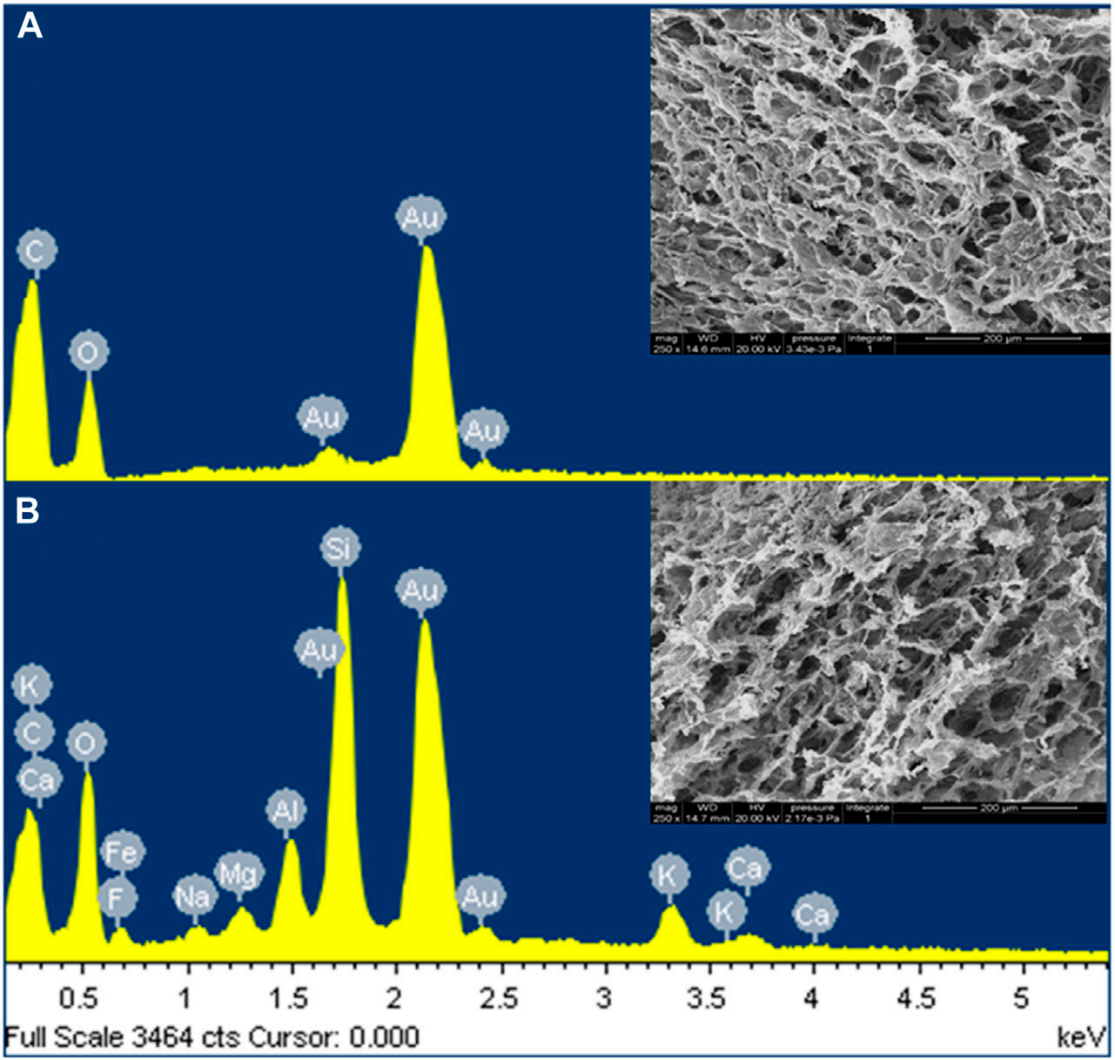
The EDX analysis and SEM images of **(A)** PVA and **(B)** PVA/0LCN-33.3SM.

The morphological and elemental analyses of the PLSM hydrogels were shown in Figure 5. A microfibrillar structure was observed in PLSM hydrogels. There was homogeneity in the PLSM hydrogel pores, with pore sizes of 40 μm. This porous structure facilitated the swelling of the hydrogels and the diffusion of MB into the interior of the hydrogels. In particular, this structure provided a large specific surface area and abundant active sites, which facilitated the adsorption of MB (Thakur et al., 2017). As can be seen in Figure 5, elemental analysis performed by EDX indicated that the MMT and SA were inserted into the pores of the hydrogel. Moreover, the increased metallic element content was confirmed to be SA embedded in the PLSM hydrogels, suggesting that they were more abundant binding sites in the hydrogels conducive to MB adsorption.

### 3.3 Absorbing capacity of the PCAM hydrogels

**FIGURE 6:**
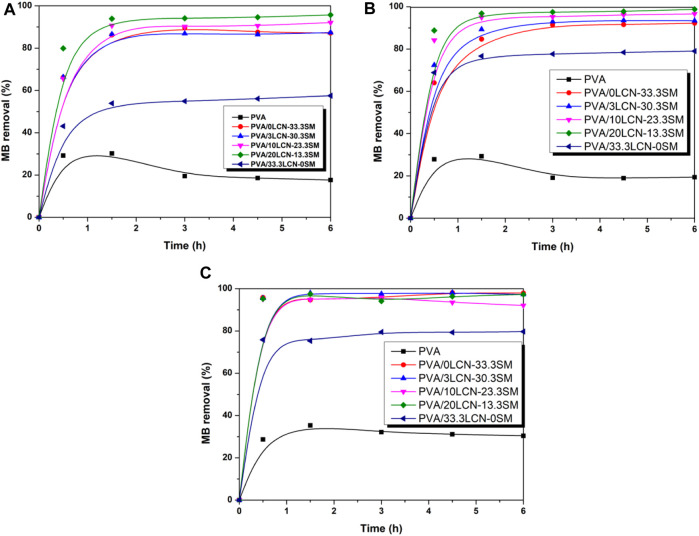
Influence of adsorption time on the removal efficiency (R%) of the hydrogels toward 0.25 g/L of MB at **(A)** 30°C, **(B)** 40°C and **(C)** 50°C.

The adsorption efficiency of 0.5 g adsorbent on 0.25 g/L methylene blue (MB) at a contact time of 6 h was investigated. The removal efficiencies of different PCAM hydrogels for MB were shown in Figure 6. The results suggested that the PVA/20LCN-13.3SM sample had the best adsorption capacity and removal efficiency. This could be due to the synergistic effect of the PVA/20LCN-13.3SM sample combined with LCN and SM, thus enhanced adsorption of pollutants by encapsulated LCN and SM was achieved as a result of this synergistic interaction. The MB removal of all hydrogels was quite pronounced at the beginning of 1.5 h and then the slow kinetics persisted until equilibrium. Compared with the adsorption hydrogel prepared by Luo et al., the adsorption efficiency of the hydrogels was higher (Luo et al., 2021). This may be due to the existence of SA which made the crosslinking network of hydrogels loose, which was conducive to the combination of hydrogels and MB.

At different temperatures, hybrid hydrogels removed MB differently as shown in Figures 6A–C. The variation was similar to the kinetic curve reported previously by other literature (Zhu et al., 2023). In spite of the increase in temperature, the removal of MB by hydrogel did not increase significantly. However, the adsorption time was significantly shortened. This could be attributed to an increase in mass transfer driving force (Zhang et al., 2018). The PVA/20LCN-13.3SM sample was adsorbed at 50°C for approximately 1 h, and the removal of MB reached 97.3%. In response to an increase in LCN mass ratio, the adsorption performance decreased. PCAM hydrogel removed only 79.7% of MB when the LCN content reached 33.3%. The reason could be that MB binds less strongly in LCN than SM; therefore, LCN is not conducive to MB adsorption. It could also be concluded that removing MB with high SM content of hydrogels under high temperatures led to a loosening of the cross-linked hydrogel network, which can rapidly adsorb MB. MMT is the main component of SM that contributed to its high absorbability. The sandwich structure of MMT consists of two tetrahedral layers sandwiched between an edge-bridged octahedral sheet combined with the 3D porous structure that ensures good adsorption performance by providing a large specific surface area. Moreover, the SA fraction contained metal elements (K^+^, Na^+^, Ca^2+^, Mg^2+^), promoting good adsorption effect on MB. Hybrid hydrogels could, however, be mechanically compromised due to the high content of SM. Hence the durability of the product should be investigated further in future work.

### 3.4 Sorption kinetics of the PCAM hydrogels

An analysis of pseudo first- and second-order kinetic equations of linear form was conducted to study the rate of MB adsorption at 40°C. The kinetic profiles of adsorption were determined during MB adsorption on the hydrogel samples. It was evident that the system was related to the model based on the *R* ^2^ of the data with this equation.

Equation of Pseudo-first-order was calculated by Eq. 4

$$\ln\left(q_{e}-q_{t}\right)=\ln q_{e}-\frac{k_{1}}{t}$$ (4)

Equation of Pseudo-second-order was determined by using Eq. 5

$$\frac{t}{q}=\frac{1}{k_{2}{q_{e}}^{2}}+\frac{t}{q_{e}}$$ (5)

Where q_t_ denotes the MB adsorbed capacity (mg/g) at time (t) in hour, k_1_ and k_2_ denote the pseudo-first- and the pseudo-second-order rate constant of adsorption (g/mg h), respectively, while q_e_ represents the equilibrium sorption amount (mg/g).

**FIGURE 7:**
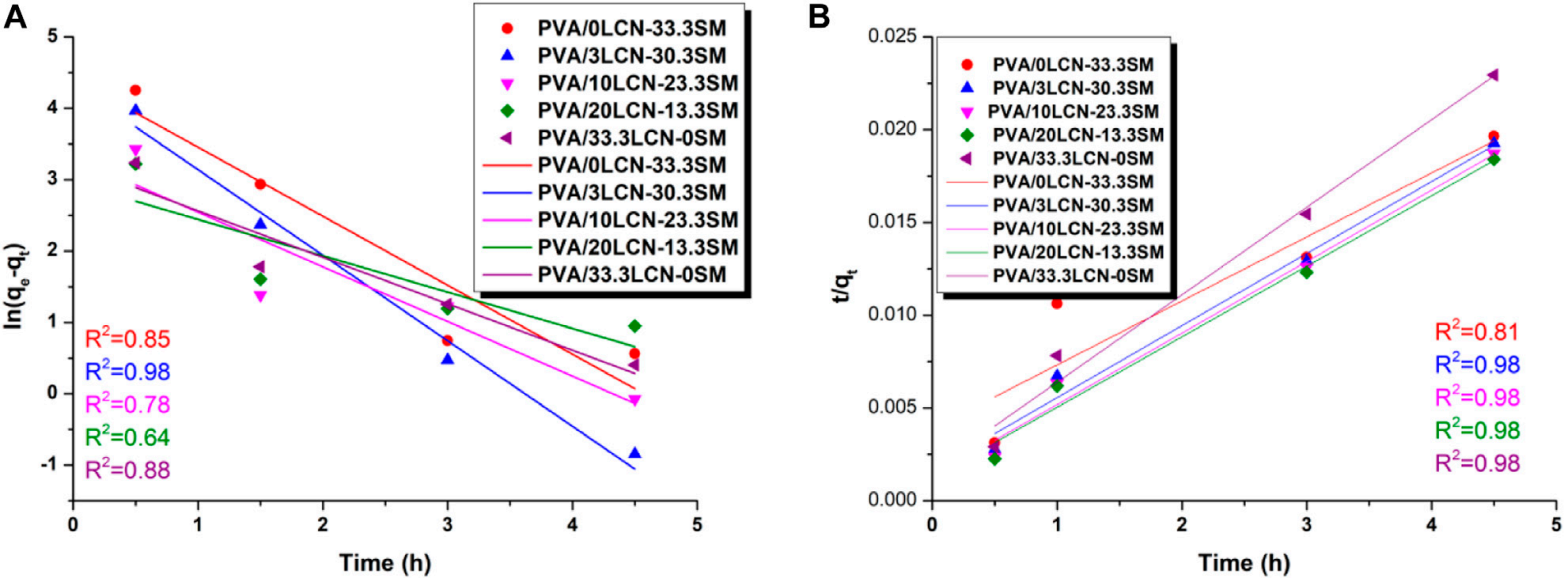
Pseudo-first-order **(A)** and second-order kinetics **(B)** of MB by the hydrogels at 40°C.

The pseudo first- and second-order kinetic equations of linear form were used to fit this adsorption process as shown in Figure 7. The coefficient of determination of the pseudo-first-order rate constant K_1_ was calculated to be greater than that of the pseudo-second-order kinetic constant K_2,_ and the opposite was true for q_e_. This phenomenon was similar to other literature reports (Wang et al., 2022). Moreover, the *R* ^2^ values of the pseudo second-order kinetic model were higher than those of the pseudo first-order kinetic model, indicating that the pseudo-second order model was better suited to describe the kinetics of MB dye adsorption on hydrogels (Figures 7A, B). Thus, these findings support that the adsorption rate of MB dye on hydrogels strongly depends on the concentration of MB and hydrogel as well as time (Melo et al., 2018; Sadik et al., 2020). As seen in Figure 7B, the adsorption of MB by PCAM hydrogels at 40°C was consistent with pseudo-second-order kinetics indicating that this adsorption process was a composite multilayer adsorption process. This process was also influenced by the diffusion of the pore network (Bulut et al., 2007; Hubbe et al., 2019).

## 4 Conclusion

Without the addition of initiators or chemicals, PVA hydrogels with excellent mechanical and adsorption properties were successfully prepared. The mechanical properties of the hybrid hydrogel were altered upon incorporating LCN and SM into the PVA water-based system. Hydrogels containing a high proportion of SM exhibited lower mechanical properties. Due to the low molecular weight of SA in SM, there was less interaction between SM and PVA. Nevertheless, excessive SM content decreased adsorption efficiency. Accordingly, PVA/0LCN-33.3SM can effectively remove 87.1% of MB in 6 h. Lignin did not decrease hydrogel adsorption efficiency, however, and may even increase it. Furthermore, Lagergren’s quasi-two-stage adsorption model was found to explain the removal of MB during the absorption tests, and the results indicated that the adsorption process had taken place on a non-uniform surface. It has therefore been demonstrated in the findings of this study that lignocellulosic materials can be utilized more effectively in wider applications.

## Data Availability

The original contributions presented in the study are included in the article/supplementary material, further inquiries can be directed to the corresponding authors.
